# α-Ionone Protects Against UVB-Induced Photoaging in Human Dermal Fibroblasts

**DOI:** 10.3390/molecules24091804

**Published:** 2019-05-09

**Authors:** Tao Tong, Jinju Park, Youna Moon, Wesuk Kang, Taesun Park

**Affiliations:** Department of Food and Nutrition, Brain Korea 21 PLUS Project, Yonsei University, 50 Yonsei-ro, Seodaemun-gu, Seoul 03722, Korea; tongtao1028@163.com (T.T.); jeanzu@naver.com (J.P.); merryouna@nate.com (Y.M.); wesuk42@naver.com (W.K.)

**Keywords:** α-ionone, dermal fibroblasts, photoaging, collagen, hyaluronic acid

## Abstract

Ultraviolet (UV) light-induced wrinkle formation is a major dermatological problem and is associated with alteration in collagen. Here, we investigated the potential of α-ionone, a naturally occurring aromatic compound, in regulation of UVB-induced photoaging in human Hs68 dermal fibroblasts and identified the mechanisms involved. We found that in human dermal fibroblasts, α-ionone inhibited UVB-induced loss of collagen. α-Ionone upregulated the molecules participating in the TGF-β–SMAD pathway (TGF-β1, phospho-SMAD2/3, *Col1A1*, and *Col1A2*), but downregulated the molecules involved in the MAPK–AP-1 signaling pathway (phospho-p38, phospho-JNK, phospho-ERK, phospho-c-Fos, phospho-c-Jun, *MMP1*, *MMP3*, and *MMP9*), in human dermal fibroblasts. α-Ionone treatment also increased hyaluronic acid contents, and this effect was accompanied by an upregulation of mRNA expression of genes (*HAS1* and *HAS2*) involved in hyaluronic acid synthesis. Thus, α-ionone is effective in the prevention of UVB-induced decrease of collagen and hyaluronic acid in human dermal fibroblasts. We propose that α-ionone may prove beneficial for the prevention of UV-induced wrinkle formation and skin damage.

## 1. Introduction

Collagen represents a superfamily of extracellular structural proteins with various physiological roles, such as the promotion of cell migration, cell growth and differentiation, and tissue development [1]. In the dermis, collagen is by far the most abundant extracellular matrix (ECM) protein and constitutes the bulk of skin (90% of dry weight) [2]. Procollagen is synthesized by dermal fibroblasts and secreted into the extracellular space, where it is enzymatically processed into collagen. Mature collagen spontaneously forms fibrils, which are largely responsible for the skin’s resilience and strength [3]. Alterations in collagen are thought to be responsible for wrinkle formation in aged skin [4]. Both intrinsic (e.g., ethnicity and anatomical variations) and extrinsic factors (e.g., UV radiation, smoking, diet, chemicals, and trauma) cause alterations in dermal collagen via two primary pathways: (1) inhibition of procollagen biosynthesis, leading to a decrease of collagen, and (2) promotion of collagen breakdown, leading to disorganized, fragmented collagen [3,5]. Thus, it is believed that in aged skin restoration of the collagen deficiency by the stimulation of new collagen biosynthesis and by a reduction in collagen degradation may be a strategy for preventing and treating the clinical manifestations of wrinkles [6].

Among the regulators influencing ECM composition, transforming growth factor β (TGF-β) is thought to be a critical mediator of ECM neosynthesis, because TGF-β modulates the expression of key components of ECM network, such as the fibrillar collagens [7]. For example, an inducible conditional deletion of TGF-β type II receptor (TβRII) in dermal fibroblasts reduces collagen deposition in the skin of mice [8]. On the other hand, it is widely accepted that mitogen-activated protein kinase (MAPK) is a key player in the degradation of ECM constituents [9]. When the MAPK signaling pathway is activated, downstream targets of this signaling cascade such as JNK, p38, and ERK are phosphorylated, and transcription factors such as AP-1 are activated, which together stimulate the expression of matrix metalloproteinases (MMPs) [10]. Both downregulation of the TGF-β signaling pathway and upregulation of MAPK signaling have been observed in dermal fibroblasts exposed to various extrinsic factors including UV irradiation [11,12,13].

Recently, we used a cell-based approach to look for small molecules that could promote procollagen production. Via this process, we found that treatment with α-ionone, an aroma compound found in herbs, fruits, roasted almonds, carrots, and raspberries [14,15], led to a robust increase in procollagen content of human dermal fibroblasts. α-Ionone is approved for use as a flavoring agent by the FDA in accordance with 21 CFR 172.515 and is granted the Generally Recognized as Safe status (GRAS) by the Flavor and Extract Manufacturers Association [14]. Although α-ionone is widely used as a food flavoring in candy, baked goods, ice cream, and beverages [14,16], research on the physiological roles of α-ionone is limited. Here, we aimed to test whether α-ionone attenuates UVB-induced photoaging in human dermal fibroblasts, and if so, to delineate the underlying mechanism.

## 2. Results

### 2.1. α-Ionone Has No Toxicity to UVB-Exposed Human Hs68 Dermal Fibroblasts

**Figure 1:**
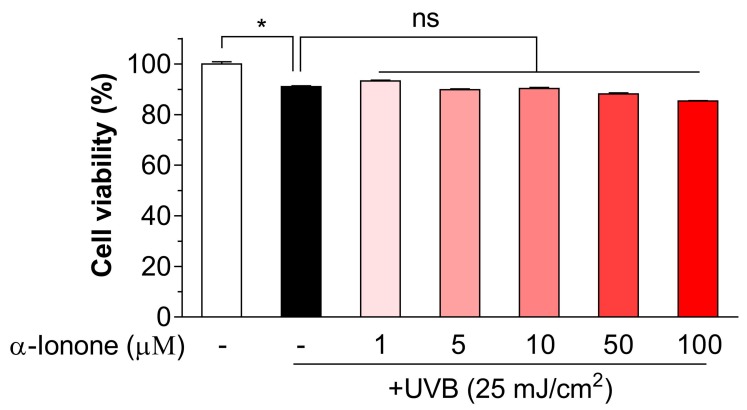
α-Ionone has no toxicity to UVB-exposed human dermal fibroblasts. A cytoprotective effect of α-ionone was determined by the 3-(4,5-dimethylthiazol-2-yl)-2,5-diphenyltetrazolium bromide (MTT) assay. Cell viability (%) was calculated as follows: (A570 of treated cells/A570 of untreated cells) × 100, where A570 is absorbance at 570 nm. Values are presented as means ± SEM (n = 3). Significant differences between groups are indicated by asterisks: * *P* < 0.05; ns, not significant (*P* > 0.05).

The effect of α-ionone on cell viability was investigated in 3-(4,5-dimethylthiazol-2-yl)-2,5-diphenyltetrazolium bromide (MTT) assays. We found that in MMT assays, UVB irradiation at a dose of 25 mJ/cm^2^ resulted in a decreased cell viability compared to non-irradiated cells (Figure 1). α-Ionone—up to a concentration of 100 µM—was confirmed to show no toxicity toward UVB-exposed human dermal fibroblasts (Figure 1).

### 2.2. α-Ionone Attenuates UVB-Induced Loss of Collagen in Human Hs68 Dermal Fibroblasts

**Figure 2:**
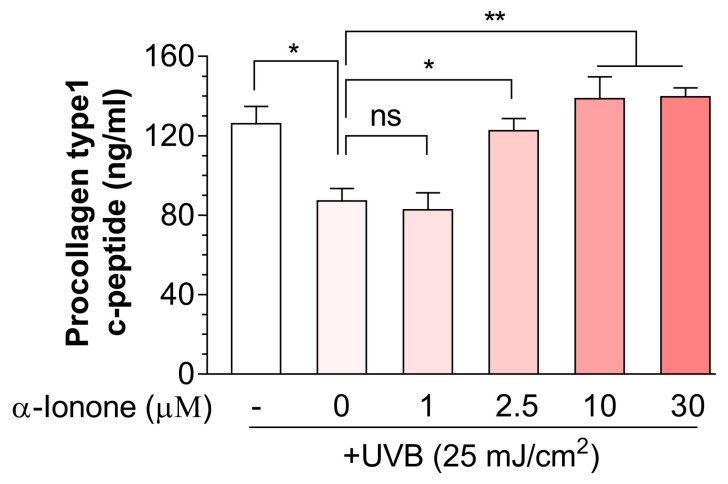
α-Ionone increases collagen contents in human Hs68 dermal fibroblasts. Values are presented as means ± SEM (n = 3). Significant differences between groups are indicated by asterisks: * *P* < 0.05; ** *P* < 0.01; ns, not significant (*P* > 0.05).

We then tested whether α-ionone could alleviate the UVB-induced collagen loss in human dermal fibroblasts. α-Ionone markedly reversed the UVB-induced decrease of procollagen I C-terminal peptide in the culture supernatants of human Hs68 dermal fibroblasts in a concentration-dependent manner, and this effect was saturated near 10 µM (Figure 2). We therefore used 10 μM α-ionone for all subsequent cell experiments.

### 2.3. α-Ionone Upregulates the Expression of Molecules Related to Collagen Synthesis in Human Hs68 Dermal Fibroblasts

**Figure 3:**
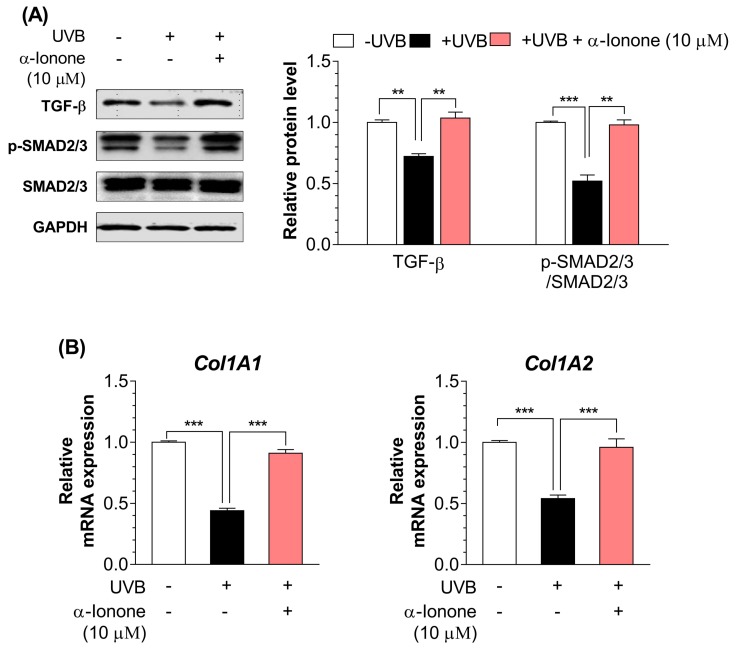
α-Ionone increases the expression of molecules related to collagen synthesis in human Hs68 dermal fibroblasts. (**A**) Protein expression of TGF-β, phospho-SMAD2/3, total SMAD2/3. (**B**) mRNA expression of *Col1A1* and *Col1A2*. Values are presented as means ± SEM (n = 3). Significant differences between groups are indicated by asterisks: ** *P* < 0.01; *** *P* < 0.001.

Next, we determined whether α-ionone treatment would modulate the expression of molecules participating in collagen synthesis in UVB-irradiated dermal fibroblasts. UVB-irradiated human dermal fibroblasts had lower protein amounts of TGF-β1 and phospho-SMAD2/3 (Figure 3A) and mRNA expression of *Col1A1* and *Col1A2* (Figure 3B) than non-irradiated cells. α-Ionone (10 µM) significantly upregulated the protein amounts of TGF-β1 and phospho-SMAD2/3 (Figure 3A), and the mRNA expression of *Col1A1* and *Col1A2* in UVB-exposed dermal fibroblasts (Figure 3B).

### 2.4. α-Ionone Suppresses the Expression of Molecules Related to Collagen Degradation in Human Hs68 Dermal Fibroblasts

**Figure 4:**
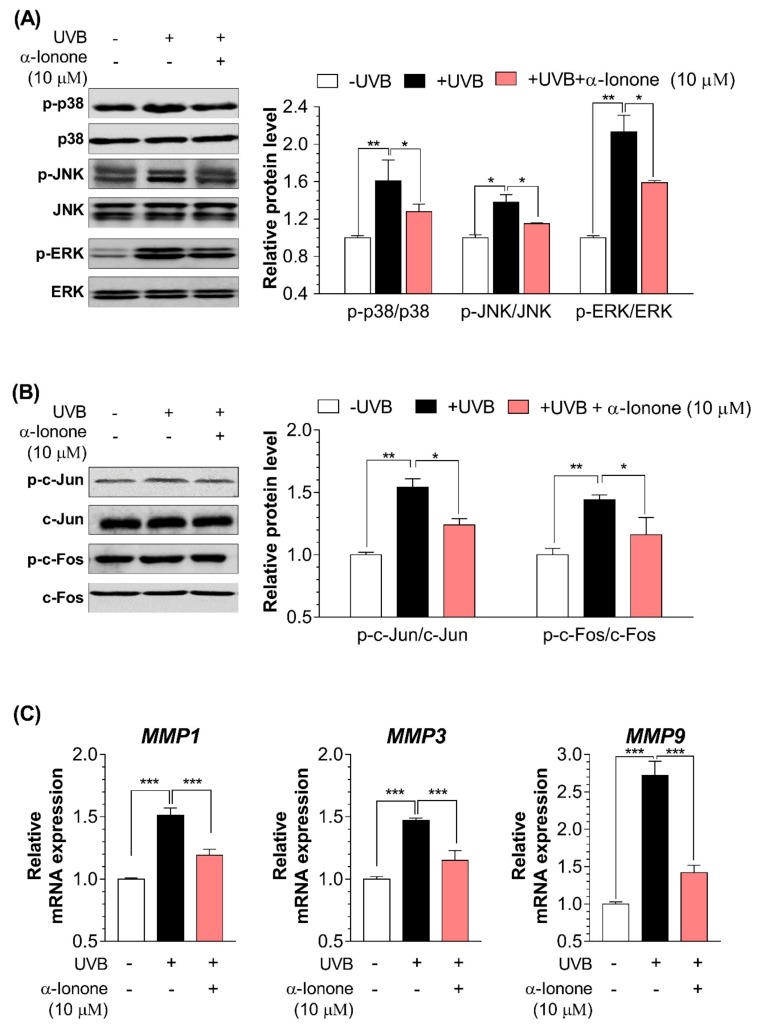
α-Ionone decreases the expression of molecules related to degradation in human Hs68 dermal fibroblasts. (**A**) Protein amounts of phospho-p38, p38, phospho-JNK (Jun *N*-terminal kinase), total JNK, phospho-ERK (extracellular signal-related kinase), and total ERK. (**B**) Protein expression of phospho-c-Jun, c-Jun, phospho-c-Fos, and c-Fos. (**C**) mRNA expression of *MMP1*, *MMP3*, and *MMP9*. Values are presented as means ± SEM (n = 3). Significant differences between groups are indicated by asterisks: * *P* < 0.05; ** *P* < 0.01; *** *P* < 0.001.

Irradiation of human dermal fibroblasts with UVB significantly upregulated the protein amounts of phospho-p38, phospho-JNK, phospho-ERK, phospho-c-Jun, and phospho-c-Fos (Figure 4A,B), and increased mRNA expression of *MMP1*, *MMP3*, and *MMP9* (Figure 4C). Treatment of human Hs68 dermal fibroblasts with α-ionone resulted in inactivation of the MAPK–AP-1 signaling pathway, as evidenced by a decreased protein amounts of phospho-p38, phospho-JNK, phospho-ERK, phospho-c-Jun, and phospho-c-Fos (Figure 4A,B), and decreased mRNA expression of *MMP1*, *MMP3*, and *MMP9* (Figure 4C).

### 2.5. α-Ionone Attenuates UVB-Induced Loss of Hyaluronic Acid (HA) in Human Hs68 Dermal Fibroblasts

**Figure 5:**
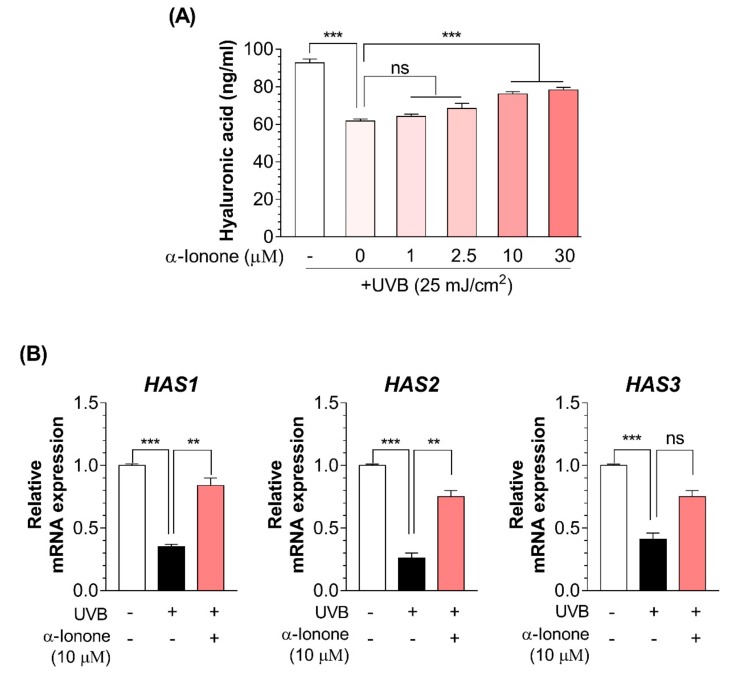
α-Ionone increases hyaluronic acid (HA) contents in human Hs68 dermal fibroblasts. (**A**) HA contents. (**B**) mRNA expression levels of genes related to HA synthesis in Hs68 cells. Values are presented as means ± SEM (n = 3). Significant differences between groups are indicated by asterisks: ** *P* < 0.01; *** *P* < 0.001; ns, not significant (*P* > 0.05).

UVB irradiation significantly decreased the HA secretion (Figure 5A) and the mRNA expression of genes (*HAS1* and *HAS2*) involved in HA synthesis in human dermal fibroblasts (Figure 5B). The stimulating effect of α-ionone on HA secretion was also concentration-dependent and reached the maximum at 10 µM (Figure 5A). Cells exposed to α-ionone (10 µM) showed an increase in the mRNA expression of genes (*HAS1* and *HAS2*) involved in HA synthesis as compared to controls (Figure 5B).

## 3. Discussion

According to the UV-induced skin damage, the UV spectrum is divided into UVA (320–400 nm) and UVB (290–320 nm) [17]. Although both UVA and UVB can interact with endogenous chromophores and photosensitizers leading to the generation of ROS causing damage to lipids, proteins, and DNA, only UVB can directly interact with DNA and produce dipyrimidine photoproducts, such as pyrimidone photoproducts and cyclobutane pyrimidine dimers [18,19]. The more harmful UV type, UVB, causes transient inflammatory reactions and sunburn acutely and induces remodeling of the skin in the long term, ultimately resulting in the symptoms of photoaging including wrinkling, pigmentation, new vessel formation, decreased turgidity, and less elasticity [20,21]. In the present study, we found that in human dermal fibroblasts α-ionone treatment reduces UVB exposure-induced loss of collagen (Figure 2).

**Figure 6:**
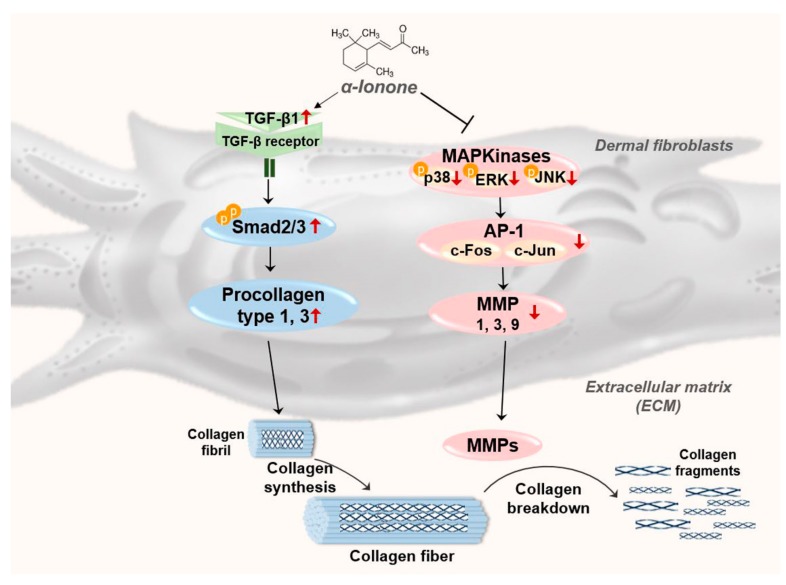
A schematic diagram illustrating the proposed mechanism by which α-ionone improves photoaging in dermal fibroblasts.

In the present study, we found that α-ionone upregulates the molecules involved in TGF-β–SMAD signaling pathway, but downregulates the molecules related to MAPK–AP-1 signaling in Hs68 cells (Figure 6). In human dermal fibroblasts, it is known that TGF-β initiates signaling via binding to and bringing together type I and type II receptor serine/threonine kinases on the cell surface. These events allow receptor II to phosphorylate the receptor I kinase domain, which then propagates the signal via phosphorylation of SMAD proteins [22,23]. These activated SMAD complexes relocate into the nucleus, where they interact with SMAD-binding elements in the promoter regions of TGF-β target genes including multiple collagens [23,24]. In addition, TGF-β is known to downregulate ECM-degrading MMPs and to upregulate plasminogen activator inhibitor 1 and tissue inhibitor of metalloproteases, which inhibit MMP activation [24]. These data indicate that the TGF-β–SMAD signaling pathway not only enhances ECM gene expression but also inhibits ECM degradation. On the other hand, it is also known that AP-1 [a menagerie of dimers of basic region-leucine zipper (bZIP) proteins that are typically composed of c-Jun and c-Fos] regulates collagen homeostasis via modulation of both collagen synthesis and degradation [25]. The activity of heterodimeric AP-1 is largely induced by signaling via the MAPK pathway and regulates the expression of several MMPs that collectively degrade the ECM, e.g., MMP1, MMP3, and MMP9. AP-1 does this by binding to its recognition sites in the promoter regions of MMP family genes [26,27]. Moreover, AP-1 is known to inhibit procollagen gene expression by blocking TGF-β type II receptor–SMAD signaling [24,28].

Fragmentation of collagen is a critical step that promotes wrinkle formation and is caused by excessive expression levels and activity of MMPs in response to UVB irradiation. At present, the MMP gene family consists of 25 members, 19 of which are found in normal skin [29]. Chronic repeated exposure to UVB is proved to elevate the expression of at least three MMPs in human skin in vivo: that is, interstitial collagenase (MMP1), stromelysin-1 (MMP3), and gelatinase (MMP9) [30,31,32]. MMP1 initiates cleavage of fibrillary collagen, typically type I and III collagens in the skin, at a single site within the central triple helix of collagen. Once cleaved by MMP1, fibrillar collagen can be further degraded by elevated amounts of MMP3 and MMP9 [3]. The combined actions of MMP1, −3, and −9 can degrade most of the proteins that constitute the dermal ECM [3]. In the current study, the mRNA expression of these three major makers (*MMP1*, *MMP3*, and *MMP9*) were significantly reduced by α-ionone treatment in human Hs68 dermal fibroblasts (Figure 4C).

In the present study, both collagen and HA increased in human dermal fibroblasts treated with α-ionone. The key molecule that contributes to skin hydration is HA, an unbranched polymeric carbohydrate consisting of alternating disaccharide units [d-glucuronic acid β(1–3)-d-*N*-acetyl-glucosamine β(1–4)] [33]. HA is synthesized at the plasma membrane by HA synthases 1–3 (HAS1–3), which assemble activated UDP-glucosamine and glucuronic acid and extrude the growing HA polymer into the extracellular compartment [34]. HAS2 appears to be the predominant isoform in the dermal fibroblasts, according to the results of quantitative real-time RT-PCR and the fact that the extent of HAS2 downregulation correlates with a decrease in HA secretion [35]. Loss of HA is thought to be a dramatic histochemical change observed in the skin exposed to UVB [36,37]. Although the factors that govern the decline of HA synthesis in the course of actinic aging are largely unknown, collagen fragment is believed to be a major contributor to the loss of HA in skin fibroblasts in response to UVB [35,38]. Degraded collagen fragments activate αvβ3-integrins and in turn suppress Rho kinase signaling and nuclear translocation of phospho-ERK, thereby leading to decreased HAS2 expression [35].

## 4. Materials and Methods

### 4.1. Reagents

Antibodies against the following proteins were obtained from Cell Signaling Technology (Danvers, MA, USA): p38 (cat. #9212; 1:1,000 dilution), phospho-p38 (#4511; 1:1,000 dilution), Jun *N*-terminal kinase (JNK, #9252; 1:1,000 dilution), phospho-JNK (#9251; 1:1,000 dilution), extracellular signal-related kinase (ERK, #4696; 1:1,000 dilution), phospho-ERK (#4370; 1:1,000 dilution), c-Fos (#4384; 1:1,000 dilution), phospho-c-Fos (#5348; 1:1,000 dilution), c-Jun (#9165; 1:1,000 dilution), phospho-c-Jun (#9164; 1:1,000 dilution), SMAD family members 2 and 3 (SMAD2/3, #5678; 1:1,000 dilution), phospho-SMAD2/3 (#8828; 1:1,000 dilution), glyceraldehyde 3-phosphate dehydrogenase (GAPDH, #2118; 1:5,000 dilution), and tubulin (#2148; 1:5,000 dilution). A horseradish peroxidase–conjugated anti-rabbit IgG antibody (1:5,000 dilution) purchased from Santa Cruz Biotechnology (Santa Cruz, CA, USA) was used as secondary antibody. DMSO (cat. #D2650), an MTT solution (#M2128), and α-ionone (#W259403) were provided by Sigma-Aldrich (St. Louis, MO, USA).

### 4.2. Cell Culture

Hs68 cells were obtained from American Type Culture Collection (Manassas, VA, USA). The cells were cultured in DMEM (HyClone Laboratories, Logan, UT, USA) supplemented with 10% of fetal bovine serum (FBS; HyClone Laboratories), streptomycin (75 mg/mL), and penicillin (120 U/mL) at 37 °C in a humidified atmosphere containing 5% of CO2. For subculturing, the Hs68 cells were washed with phosphate-buffered saline (PBS), and a 0.25% trypsin/ethylenediaminetetraacetic acid solution was used to detach the cells.

### 4.3. The Cell Viability Assay

Hs68 cells were seeded at density 2 × 10^4^/mL in a 96-well plate and cultured overnight. Then, the cells were cultured with various concentrations of α-ionone (1, 5, 10, 50, or 100 μM) for 24 h. After that, the cells were exposed to UVB (25 mJ/cm^2^) and then incubated with different concentrations of α-ionone for 24 h in serum-free DMEM. UVB irradiation was conducted using a CL-1000M UV crosslinker (UVP, Upland, CA, USA) with a UV peak at 302 nm. Next, 3-(4,5-dimethylthiazol-2-yl)-2,5-diphenyltetrazolium bromide (MTT) solution (50 μL, 5 mg/mL, Sigma-Aldrich) was added into each well, and the plates were incubated at 37 °C for 4 h. After that, DMSO (50 μL) was added into each well and incubated at room temperature for 30 min. Cell viability was evaluated via determining mitochondria-dependent conversion of yellow tetrazolium salt MTT to purple formazan crystals by metabolically active cells. The microplate reader (Versa Max, Molecular devices, Sunnyvale, CA, USA) was used to evaluate the optical density at 570 nm [39].

### 4.4. Determination of Procollagen I C-Terminal Peptide and HA Secretion

The cells were cultured at a density of 105 cells/well in a 24-well plate and pretreated with α-ionone (1, 2.5, 10, or 30 μM) for 24 h. The cells were next washed with PBS and were exposed to UVB (25 mJ/cm^2^) through a thin layer of PBS. After that, Hs68 cells were cultured with serum-free DMEM containing α-ionone (1, 2.5, 10 or 30 μM). The culture medium was collected after 24 h, and then type I procollagen content was determined by means of the Procollagen Type I C-Peptide Enzyme Immunoassay Kit (MK101; Takara, Shiga, Japan). HA in fibroblast culture supernatants was quantified by the Hyaluronic acid Quantikine^®^ ELISA kit (DHYAL0, R&D Systems, Minneapolis, MN, USA).

### 4.5. RNA Extraction and PCR

Total RNA was extracted from human Hs68 dermal fibroblasts using the TRIzol Reagent (Life Technology, Carlsbad, CA, USA). Complementary DNA synthesis was conducted with dithiothreitol (0.1 M), total RNA (1 μg), dNTP (2.5 mM), RNase inhibitor (40 U/μL), 5× RT buffer diluted to 1× and reverse transcriptase (200 U/μL) in a total reaction volume of 40 μL at 37 °C for 2 h. After reverse transcription of RNA, quantitative PCR was conducted with the iQ SYBR Green Supermix (Bio-Rad, Hercules, CA, USA) on a CFX Connect™ Real-Time PCR Detection System (Bio-Rad). Primer sequences used for PCR are listed in Table 1. The results on the optical density ratio of a target gene to GAPDH are presented as mean ± SEM of at least three independent experiments.

### 4.6. Protein Extraction and Western Blotting

The cells were homogenized in lysis buffer (2 μg/mL aprotinin, 5 mM EDTA, 1 μg/mL leupeptin, 50 mM NaF, 100 mM orthovanadate, 50 mM sodium pyrophosphate, 1 mM phenylmethanesulfonyl fluoride, 1 μg/mL pepstatin A, 1% Triton X-100, and 100 mM Tris-HCl pH 7.4). Equal amounts of protein samples (40 μg) were boiled for 5 min and separated by SDS-PAGE in an 8–12% gel. Next, the resolved proteins were transferred to a nitrocellulose membrane (Whatman, Dassel, Germany). After blockage with 5% bovine serum albumin, the membranes were incubated with primary antibodies overnight at 4 °C, followed by the corresponding secondary antibodies. ECL Chemiluminescent Detection Reagent (GE Healthcare, Buckinghamshire, UK) was used to visualize the protein bands. After that, the band images were obtained using a WSE-6100 LuminoGraph system (ATTO, Tokyo, Japan). The results on the ratio of signal density of target proteins to that of either GAPDH or tubulin are presented as mean ± SEM of at least three independent experiments.

### 4.7. Statistical Analysis

Data are presented as mean ± standard error of mean (SEM). For statistical analyses, significance of the differences between two groups was determined using Student’s t test. GraphPad Prism 7 software (GraphPad, San Diego, CA, USA) was used to perform statistical analyses. In all statistical tests, significance was set at * *P* < 0.05, ** *P* < 0.01, and *** *P* < 0.001.

## 5. Conclusions

We demonstrated that α-ionone could attenuate UVB-induced loss of collagen and HA. This study highlights potential usefulness of α-ionone for the treatment of wrinkles and skin dryness associated with various (pathological) physiological states. If α-ionone works in human skin tissues the way that it does in human dermal fibroblasts model, the outcome could be an effective therapeutic approach for the prevention or treatment of photoaging or other pathologies related to photoaging. Additional research is required to further determine the potential for developing α-ionone as a pharmacologic strategy for improving the density and integrity of the collagen network and skin hydration.

## Figures and Tables

**Table 1:** Primer sequences.

Type	Gene Description	Sequences (5′→3)
Human	>Collagen type I alpha 1 chain (*Col1A1*)	F: TTGCTCCCCAGCTGTCTTAT
		R: AGACCACGAGGACCAGAGG
	Collagen type I alpha 2 chain (*Col1A2*)	F: CGGAGGTATGCAGACAACGA
		R: CTAGGGTGCCTCCAAAAGGG
	Matrix metalloproteinase 3 (*MMP1*)	F: ATTCTACTGATATCGGGGCTTTGA
		R: ATGTCCTTGGGGTATCCGTGTAG
	Matrix metalloproteinase 3 (*MMP3*)	F: TGAGGACACCAGCATGAACC
		R: ACTTCGGATGCCCAGGAAAG
	Matrix metalloproteinase 3 (*MMP9*)	F: TTGACAGCGACAAGAAGTGG
		R: GCCATTCACGTCGTCCTTAT
	Hyaluronan synthase 1 (*HAS1*)	F: TACAACCAGAAGTTCCTGGG
		R: CTGGAGGTGTACTTGGTAGC
	Hyaluronan synthase 2 (*HAS2*)	F: GTGGATTATGTACAGGTTTGTGA
		R: TCCAACCATGGGATCTTCTT
	Hyaluronan synthase 3 (*HAS3*)	F: GAGATGTCCAGATCCTCAACAA
		R: CCCACTAATACACTGCACAC
	Glyceraldehyde 3-phosphate dehydrogenase (*GAPDH*)	F: GTGAAGGTCGGAGTCAACG
		R: TGAGGTCAATGAAGGGGTC
