# Erratum: Tong, T., et al. α-Ionone Protects Against UVB-Induced Photoaging in Human Dermal Fibroblasts. *Molecules* 2019, *24*, 1804

**DOI:** 10.3390/molecules25153517

**Published:** 2020-07-31

**Authors:** Tao Tong, Jinju Park, Youna Moon, Wesuk Kang, Taesun Park

**Affiliations:** Department of Food and Nutrition, Brain Korea 21 PLUS Project, Yonsei University, 50 Yonsei-ro, Seodaemun-gu, Seoul 03722, Korea; tongtao1028@163.com (T.T.); jeanzu@naver.com (J.P.); merryouna@nate.com (Y.M.); wesuk42@naver.com (W.K.)

The authors wish to make the following change to their paper [1]. The Funding section is incorrect in the published paper. The correct Funding should be as follows:

This work was supported by the National Research Foundation of Korea (NRF) grant funded by the Korea government (MSIT) (No. 2019R1A2C2003340) and by Innopolis Foundation through the Technology Commercialization Project, funded by Ministry of Science and ICT (2018-JB-RD-0035). 

We apologize for any inconvenience caused to the readers by this mistake.
